# Moral uncertainty in bioethical argumentation: a new understanding of the pro-life view on early human embryos

**DOI:** 10.1007/s11017-014-9309-1

**Published:** 2014-09-18

**Authors:** Tomasz Żuradzki

**Affiliations:** Institute of Philosophy, Jagiellonian University, ul. Grodzka 52, 31-044 Kraków, Poland

**Keywords:** Human embryo research, Moral status, Pro-life view, Decision theory, Normative uncertainty

## Abstract

In this article, I present a new interpretation of the pro-life view on the status of early human embryos. In my understanding, this position is based not on presumptions about the ontological status of embryos and their developmental capabilities but on the specific criteria of rational decisions under uncertainty and on a cautious response to the ambiguous status of embryos. This view, which uses the decision theory model of moral reasoning, promises to reconcile the uncertainty about the ontological status of embryos with the certainty about normative obligations. I will demonstrate that my interpretation of the pro-life view, although seeming to be stronger than the standard one, has limited scope and cannot be used to limit destructive research on human embryos.

## Introduction

Human embryos in the early stages of development are ontologically ambiguous entities.^1^ This ontological ambiguity of early embryos, which has been discussed by some philosophers and bioethicists [1, 2], although not recognized by the majority of pro-life followers, is an underlying reason for the uncertainty about the moral status of human embryos. What is the morally permitted way of acting in the face of uncertainty about the moral status of some beings? Does this uncertainty “cast serious doubt on the arguments claiming … full protection” [3, p. 153] of embryos, or is it the other way around: does it give a reason for acting in a cautious way and treating these entities as if they had very high or even full moral status? In this article, I will argue that the uncertainty about the moral status of the entity we are dealing with sometimes, indeed, gives us a reason to act in a precautionary way. Nevertheless, it cannot be a reason for granting unconditional full protection to embryos, which could result, for example, in banning research on human embryos or in vitro fertilization (IVF) procedures.

## The standard interpretations of the pro-life views

The dominant view in recent Western moral sensibility is that human embryos have a special status, but not as high as that of children or adults. This means that the embryos’ right to life (especially in the early stages of development) can be weighed against some individual or societal benefits in a way that the right to life of children or adults cannot be. This view is present in many societal practices (e.g., the acceptance of “morning-after pills” that destroy early embryos, not caring about massive early embryo loss, etc.) as well as in the legal regulations of many European states where destructive research on early human embryos is legally permitted if it serves important scientific purposes. In most European states research is permitted only on embryos that are no longer needed for reproduction, but in some, permission is extended to embryos created specifically for research (e.g., Belgium, Sweden, the UK) [4]. Even in those states where embryo protection seems to be much more intense and embryo research is prohibited (e.g., Austria, Ireland, Poland), embryos are not treated by the law as having full moral status or a full right to life from the very beginning (e.g., the law in these countries accepts the sacrifice of embryos during IVF procedures for individual reproductive purposes). The view that ascribes gradual moral status to embryos also has a strong historical background within the Catholic tradition, at least up until the end of the 19th century [5].

Some opponents of destructive embryonic research claim that every human embryo should be treated as having full moral status and a full right to life. If this is correct, the justification for destructive embryonic research should be similar to the justification that permits killing a human adult. But there is full agreement in medical ethics that it is morally wrong (and that it should not be legally permitted) to intentionally kill an adult human being in order to pursue medical research (as well as for any reason other than self-defense). Moreover, for the supporters of pro-life views, destructive embryo research or IVF should be morally worse than some cases of abortion (e.g., those performed because of self-defense reasons), as these procedures result in the deliberate creation of embryos that are either destroyed for experiments or abandoned for reproductive purposes.

How should one understand the pro-life view, which claims that the moral status of embryos and adult human beings are equal? The most influential justification is the theological argument given by current Catholic doctrine. The recent instruction *Dignitas Personae* published by the Congregation for the Doctrine of the Faith in 2008 claims that “the human embryo has…, from the very beginning, the dignity proper to a person” [6]. The same position can be found in many earlier documents of the Catholic Church (e.g., in *Donum Vitae* published in 1987: “the fruit of human generation, from the first moment of its existence, that is to say from the moment the zygote has formed, demands the unconditional respect that is morally due to the human being in his bodily and spiritual totality” [7]; a very similar claim may be found in the encyclical *Evangelium Vitae* published in 1995 [8, section 60]). “The very beginning” or “the first moment” is commonly understood as the moment of fertilization (or an equivalent process, e.g., nuclear transfer). What does it mean to have “the dignity proper to a person”? The Catholic Church adopts a standard definition of a person proposed by Boethius: “an individual substance of a rational nature” (*naturae rationalis individua substantia*). One contemporary Thomist interprets this formula as follows: “a person must consist of one, ongoing ontology and a soul which endows it with the powers of rationality, i.e., intellect” [9, p. 140]. These three requirements (unicity, ontological continuity, and an intellectual soul) are necessary requirements for a being to have the status of a person.

The theological justification is important, since it is intertwined with philosophical justifications of the pro-life view (see, e.g., [10–12]). Even if some of these philosophical justifications do not appeal to theologically loaded concepts, like those of “soul” or “person,” they share with the theological justifications a commitment to substance ontology, which is the claim that “human” is “a substance concept” and an entity becomes a human substance in one discrete event [13]. This kind of approach says that human beings have some substantial properties (as opposed to accidental) that indicate what kind of beings they are. Although this position by itself does not justify the ascription of moral status (one might accept a “substance ontology” and yet think that moral status is a human projection), it is commonly used by philosophers defending the pro-life view. Substance ontology seeks to determine either the exact moment when a human being comes into existence or the process in which it emerges: before and after this there are two different entities. Moreover, each being must also be distinct from all other entities: e.g., when a human embryo splits to form identical twins, substance ontology would claim that either one being ceased to exist and that two new beings appeared in its place or that an original being survives the twinning, but a second individual emerges alongside it. Some philosophers claim that advances in biology can help to identify the moment (e.g., of the fusion of the nuclei) when a human being begins. In the opinion of those who defend the pro-life view, the process of fertilization (or some moment during it) marks when a new substance comes into being (although they can disagree whether it happens during the fusion of the sperm and egg, the fusion of the nuclei, or the first cell division). In the case of cloning, the joining of the nucleus and the enucleated egg marks this moment and therefore cloned embryos ought to be treated as having the same moral status as other human embryos [14, p. 4]. There is also an additional pragmatic reason for treating fertilization as the moment of substantial change: since embryonic development occurs along a continuum, it is impossible to make any convincing distinction at any other point in this process (this argument is also visible in the *Declaration on Procured Abortion* published in 1974: “It [a new human being] would never be made human if it were not human already” [15]). Therefore, we should accord human embryos full moral status from the moment an oocyte is fertilized or nuclear transfer takes place.

Proponents of the pro-life view who justify their views either on theological or philosophical grounds assume that the essential qualities of the intellectual soul or the human organism do not have to be actualized for a person to be present, since human embryos are organisms that, from the very beginning, have potentiality to develop into an adult human being. They adopt so-called active potentiality: the actualization of potentiality arises from the nature of the entity itself, without dependence on external causes. The distinction between active and passive potentiality helps to explain why a human embryo has full moral status but human gametes do not (they have only passive potentiality). There are also other reasons why the notion of potentiality is important: the cells in a human being do not differ in the genetic information they contain but only in the subset of the genes they express. One could ask, why do most proponents of the pro-life view not ascribe full moral status to any single human cell except the one-celled embryo? The answer could be that the fertilized egg is unique because, unlike somatic cells, it possesses an active potentiality to develop into an organism using its own genetic information [10, p. 53]. Therefore, the strict ontology and the concept of active potentiality help to maintain that it is wrong to kill an embryo, since “she is identical to an entity that, at some time later in her development, everyone agrees it is wrong to kill” [11, p. 249].

## Some problems with the standard interpretations

A well-known reason for the uncertainty about the moral status of early human embryos is the ambiguity concerning their ontological status, especially their capacity for splitting and recombination. Early embryos can be teased into two or more parts, each of which can develop into a fetus. Conversely, two different embryos may fuse and develop into one fetus and then an adult human being with cells containing two different DNAs, which sometimes happens naturally and often results in genetic disorders, but many human chimeras are completely healthy and unaware of their genetic condition. This is one reason why even some Catholic scholars claim that early human embryos cannot have souls until after the second week of fertilization, when the primitive streak begins to form. The proponents of “delayed animation” underline the fact that the moment of ensoulment must coincide with the formation of an ontological individual [16, 17]. The capacity for splitting and recombination and the possible lack of ontological continuity is also a reason why some philosophers who defend a pro-life view on abortion do not ascribe full moral status to early embryos [18].

The second reason for the uncertainty about the moral status of early human embryos is related to the high rate of their “mortality”: about 50–80 % of embryos naturally die within the first few hours or days after conception. Thus, the proponents of the pro-life view are vulnerable to the critique that they do not in fact ascribe as high a moral status to embryos as they verbally claim. If they in fact think that embryos have full moral status, why do they not care about the more than 200 million “young and innocent” beings dying every year after a very short life? Why do they not call for global research programs that could improve the embryos’ survival rate? A philosopher who has recently discussed this problem writes that “finding means of saving even 5 % of embryos from spontaneous abortion would save more lives than a cure for cancer” [19, p. 15]. It seems that everybody, including hardline pro-life thinkers, accept that procreation involves the (intentional) creation and (non-intentional, but easy to foresee) destruction of many embryos no matter if the fertilization occurs in vitro or in vivo: “a mother of three children could be expected to have also had approximately five spontaneous abortions” [19, p. 13]. It is extremely hard to justify that creating some entities and then letting them die at a very young age is permissible as part of a procreative project if these entities indeed had a moral status equal to that of an adult human being. This is why one Thomist scholar recently came to the conclusion that: “it seems odd to believe that God … would permit the needless death of so many persons. It seems more reasonable to conceive of them as naturally rejected biological material—not persons” [9, p. 156].

Thirdly, assuming the existence of a rational “soul” from the very moment of conception (and thereby the beginning of personhood) plays a much less important role within the Catholic tradition than is commonly thought [20]. The official teaching of the Catholic Church on prenatal life explicitly recognizes this philosophical uncertainty about the beginning of ensoulment, which makes many pro-lifers holier than the Pope. For example, in the *Declaration on Procured Abortion*, the Congregation states, “this declaration expressly leaves aside the question of the moment when the spiritual soul is infused. There is not a unanimous tradition on this point and authors are as yet in disagreement. For some it dates from the first instant; for others it could not at least precede nidation” [15].

This position is sustained in all official documents. For example, in 1987 in *Donum Vitae*, the Congregation, on the one hand, wrote that “the conclusions of science regarding the human embryo provide a valuable indication for discerning by the use of reason a personal presence at the moment of this first appearance of a human life,” but on the other hand, it does not confirm explicitly that embryos are persons: “The Magisterium has not expressly committed itself to an affirmation of a philosophical nature” (that is, whether early embryos have souls) [7]. This position was repeated by John Paul II in 1995 in *Evangelium Vitae* [8, sec. 60], and in 2008 by the Congregation in *Dignitas Personae,* which interprets this view explicitly: “*Donum Vitae*, in order to avoid a statement of an explicitly philosophical nature, did not define the embryo as a person” [6]. Some commentators argue that this last fragment “pull[s] back from a growing trajectory in the Church’s teaching, from debates over ensoulment to the benefit-of-the-doubt approach taken by the CDF [the Congregation] in its 1974 *Declaration on Procured Abortion* and the more robust (albeit interrogative) defense of the personhood of the embryo during the pontificate of John Paul II” [21, p. 314], but it seems that the constant avoiding of the definition of an embryo as a person by the Congregation or the successive popes is clear evidence that there is no substantial shift of views between 1974 and 2008.

Why does the Congregation claim, on the one hand, that human embryos “have dignity proper to a person” and should be treated as persons from the very moment of conception, but on the other hand, does not support “conceptionism” explicitly (i.e., the view that ensoulment takes place during conception)? How is it possible to combine certainty about normative obligations with uncertainty about ontological status, as is expressed, for example, in this fragment of the *Declaration on Procured Abortion*: “From a moral point of view this is certain: even if a doubt existed concerning whether the fruit of conception is already a human person, it is objectively a grave sin to dare to risk murder” [15]?

The standard interpretation claims that the argument from active potentiality must again appear on the stage: even if we are not sure whether an early embryo has a soul, we are certain that it is “a human life, preparing for and calling for a soul” [15] (in other Church documents an early embryo is also called: “a human being”) and thus it possesses capabilities that will be exercised later, but which make it a person now. What does it mean that an embryo is “a human life” or “a human being”? The simplest answer is that it is an organism that contains the human genome. Does it mean that the uncertainty about the moment of ensoulment does not have to coincide with uncertainty about the existence of human life? Theoretically, it is possible to be certain that some organism is “a human life,” but at the same time to be uncertain whether it has a rational soul, since this would only “prepare for” it and “call for” it. But this would not be a proper interpretation of the Catholic view. According to the Thomistic metaphysics adopted by the recent Catholic documents, all human beings are persons, and it is not possible to have the ontological status of a human life without an intellectual soul which must inform the matter of each human being [22]. Therefore, the standard interpretation does not explain the rigid stance of pro-lifers against human embryo research in the face of uncertainty about the ontological status of embryos which is recognized even within the Catholic doctrine.

## The argument from normative uncertainty (ANU)

I will argue that in the case of early embryos there exists a strong pro-life argument based on the criteria of rational action under uncertainty that can easily explain the rigid stance of pro-lifers against human embryo research even in the face of their uncertainty about the ontological status of embryos. It is strong because (1) it does not require the acceptance of Thomistic metaphysics or any other substance ontology, (2) it avoids the unsolvable discussion about the moment of ‘ensoulment’ or about the beginning of personhood, (3) it does not depend on claims about the ontological status of embryos and their identity through time with the human beings they precede, and (4) it is able to bypass the notorious problem of potentiality.

The case of early embryos I discuss here is a manifestation of the broader problem of our moral fallibility and meta-reasoning in making moral decisions [23–25]. So, this discussion may have interesting applications to other cases of normative uncertainty because even when we feel quite certain about moral issues, we are susceptible to moral mistakes, and we need to have a method to deal with the possibility that we will get some crucial moral issues wrong. For example, we have to act very often in the face of uncertainty about moral doctrines, specific moral duties, or the ontological status of some entities. In this last case, we may have our doubts because (1) we may be unsure when they begin to belong to some ontological class, or (2) they are difficult to classify, or, last but not least, (3) our ontological classifications themselves are vague. The problem is of crucial importance for ethics, since belonging to some ontological class usually has serious implications for moral status. Except for human embryos at the early stages of development, real-life examples of entities with uncertain ontological—and thus moral—status include some products (real or only possible) of genetic engineering: the “embryo-like” products of therapeutic cloning or parthenogenesis, blastomeres that can be changed in laboratory conditions into totipotent stem cells, and induced pluripotent stem cells (iPS). Some philosophers have also argued that the uncertainty about the status of animals provides a reason for acting cautiously and treating them as if they had very high moral status. Peter Singer used this type of reasoning to defend vegetarianism. He considered an example of someone who is uncertain whether killing a pig in order to eat it is right or wrong. Singer claims that usually there are no pressing moral reasons for killing animals: the fact that one might prefer a dish containing meat to a vegetarian meal is hardly a matter of great moral significance for anyone. Therefore, even if we do not accept that animals have important moral status “it would seem better to give the pig the benefit of the doubt” [26, p. 252] (cf. [27]).

The argument from normative uncertainty is visible in the Congregation documents: “it suffices that this presence of the soul be probable (and one can never prove the contrary) in order that the taking of life involve accepting the risk of killing a man, not only waiting for, but already in possession of his soul” [6, fn. 19] (cf. [7]). The structure of this argument is as follows: (1) we have to act in the face of uncertainty about the moral status of some entity (E)—either because we are not sure when it begins, or because we are not sure if it belongs to some ontological class, or because we are not sure which moral doctrine is the right one; (2) even if there are some reasons (r) to destroy E, they are not decisive; (3) if E belongs to this ontological class (or if E starts to exist at a particular moment of time or some moral doctrine is the right one), the moral status of E is full, i.e., equal to the status of an adult human being; (4) if the entity has full moral status, it is seriously wrong to destroy it and r does not justify it, whereas if it does not have such a high status we can destroy it without committing anything that is morally wrong; (5) rationality and morality require that we should act as if E had full moral status even if we do not believe that it has full moral status (I have discussed a similar argument in Polish in [28]).

The argument presupposes a form of moral fallibilism: one can never be certain that a given view on ontological or moral questions is correct or true. No matter how strong someone’s views are about the ontological status of embryos, it is always possible (“one can never prove the contrary”) that in fact early embryos have an ontological status such that we should treat them because of moral reasons as equal to adult human beings. If there is only a probability, no matter how small, that embryos have this kind of status, we should act as if they had souls indeed (“mere probability that a human person is involved would suffice to justify an absolutely clear prohibition of any intervention aimed at killing a human embryo”). Why does the exact probability not matter? The ANU makes use of the decision theory model of moral reasoning in situations of normative uncertainty, that is, in situations where we are not sure, for example, what the ontological status of the embryo is and what its moral implications are. Usually, in cases of factual risk or uncertain consequences, to determine which course of action we should pursue, the decision theory model of reasoning recommends taking the subjective probabilities of various outcomes and combining them with the values we attach to the outcomes. The ANU appeals to analogical reasoning in the face of normative uncertainty: we should evaluate the subjective probabilities of different doctrines about the moral status of early human embryos and combine them with the disvalues attached by these doctrines to the destruction of embryos. If the disvalue attached by some doctrine to the intentional destruction of an embryo is substantial (or even infinite, if we give an absolutist interpretation to doctrines that strictly prohibit intentionally killing a person or something of similar status) this parameter dominates the calculus. The probability that embryos have full moral status could be minimal (or, in other words, the doctrine assuming that embryos have full moral status could be extremely unreliable, though not false), but the disvalue of the risk of intentionally destroying a being with full moral status will prevail, because in the moment of decision, we do not know whether it indeed has this kind of status or not. This reasoning has an important implication: when we act under normative uncertainty, we should not always act according to the normative view that in our opinion is most reliable. In the case of embryo research, the ANU does not claim that anyone should believe that early embryos have full moral status from the very beginning or the ontological status of persons. Even if we strongly believe (but we cannot be sure) that it is permissible to destroy human embryos until the 14th day after conception in order to conduct important scientific research, it is rational and moral to act in a precautionary manner: any rational and moral agent should act as if early embryos had full moral status (or as if they were persons, as if they had souls, etc.) This emphasis on pragmatic reasons (on the question “What should we do under normative uncertainty?” and not “What should we believe under normative uncertainty?”) distinguishes this argument from Pascal’s wager (although, strictly speaking, Pascal did not maintain that it is rational to believe in God, but rather, that it is rational to resolve on believing in God).

It may seem that the ANU follows the traditional approach of Catholic moral theology developed in the 16th and the 17th centuries for resolving doubts [5]. This tradition makes a distinction between a doubt of fact and a doubt of law. In this first case, a decision maker is uncertain whether some particular act will or will not fulfill the law. In the second case a decision-maker is uncertain whether some particular moral rule is in effect (this approach models moral duties according to the natural law tradition: moral duties depend on objectively existing rules imposed by God and usually can be known by reason). The difference between these two approaches is crucial because Catholic moral theology used to claim that in the case of a doubt of fact always the safer course of action must be followed: we are not allowed to act if there is a probability that an innocent person will be killed or harmed. Whereas in the case of a doubt of law a decision-maker does not need certainty to act, even if human life is at risk. The dominant procedure is probabilism, which claims that a decision-maker is allowed not to follow a rule if there exists a probable opinion in favour of liberty (that is, that a particular moral rule does not have to be obeyed), even though the contrary opinion is more probable. Two other decision procedures on how to act in the case of a doubt of law are: equiprobabilism which claims that one is allowed to follow the opinion in favour of liberty only in cases when conflicting opinions are nearly equally probable; and probabiliorism which says that a decision maker may do it only when opinions in favour of liberty are more probable. Two factors were relevant to measure whether an opinion is more probable than another: the number of Church fathers supporting some opinion and their authority. Carol A. Tauer gives two examples in which probabilistic methods were accepted by the Church in the past: the castration of boys for religious choirs and the acceptance of slavery. In both cases the existence of conflicting opinions about the permissibility of these practices was taken as an argument in favor of liberty [5, p. 25].

It is easy to notice that the ANU used in the recent Catholic documents treats uncertainty about the ontological status of early embryos and the moment of ensoulment differently: as a doubt of fact, not as a doubt of law. On the one hand this is problematic in the light of the Congregation’s own statement that “it is not up to the biological sciences to make a definitive judgment on questions which are properly philosophical and moral such as the moment when a human person is constituted.” So treating the question about the moment of ensoulment as a doubt of fact by the Congregation requires adopting quite an unusual understanding of the term “fact” that would include theological facts (e.g., “embryos have souls”). On the other hand, in the case of early embryos, there is not “a doubt of law,” because the moral rule expressed by the reliable authority (the Congregation) is clear: early embryos should be treated as persons. Tauer argues that this is a third category of doubt—theoretical doubt that is equivalent to a doubt of law and thus should be handled according to probabilistic methods. In the final section, I will show why these probabilistic methods are not always the proper approach and I will point out more fundamental problems with the ANU-type of reasoning.

## The ANU in bioethical discussions

The ANU as an argument in favor of the pro-life view on the moral status of early embryos and its potential applications has been overlooked by many contemporary bioethicists, and the ontological ambiguity of early embryos (though not of the fetuses) is not widely recognized within the pro-life camp. Moreover, the ANU type of reasoning has also been recently applied to some embryo-like products of genetic engineering. In *Dignitas Personae* the Congregation claims that we should treat the products of human parthenogenesis, altered nuclear transfer, and oocyte assisted reprogramming (if someone were to carry out these procedures on human material) as human persons because there are:

… questions of both a scientific and an ethical nature regarding above all the ontological status of the “product” obtained in this way. Until these doubts have been clarified, the statement of the Encyclical *Evangelium Vitae* needs to be kept in mind: “what is at stake is so important that, from the standpoint of moral obligation, the mere probability that a human person is involved would suffice to justify an absolutely clear prohibition of any intervention aimed at killing a human embryo.” [6]

This formulation suggests that the prohibition can be extended to other “embryo-like products” that are not enumerated explicitly in this document, even if there “is the mere probability that a human person is involved.” This means that the ANU could prohibit any procedure that leads to obtaining totipotent cells (which can become an embryo under the proper conditions). For example, it could be extended to the early-stage blastomeres extracted from an embryo that might also be totipotent and, at least in theory, capable of developing into a second embryo.

Some philosophers have recently discussed a similar type of reasoning in the context of abortion [23, 29–31]. In one article by two eminent philosophers, Frank Jackson and Michael Smith, the authors claim that there is a group of Catholics who:

… oppose early stage abortion while granting that it is far from certain that an early stage foetus is a person. They argue that it is possible that early stage abortion is not the intentional killing of an innocent person because it is not the killing of a person, and, therefore, that it may not be ruled out by absolutist prohibitions against the intentional killing of innocent persons. But, all the same, early stage abortion ought not to be allowed because we cannot be sufficiently confident that the early stage foetus is not a person. Their position is, precisely, that early stage abortion may or may not be something that is objectively wrong but it is certainly something that decision-ought not to be done. [30, pp. 270–271]^2^

Their claim seems to be mistaken in the light of recent theological or doctrinal discussions by Catholics regarding the permissibility of early stage abortion. Admittedly, the Congregation claims that this kind of uncertainty about the personhood of early embryos exists, and that some scholars or theologians claim that the intellectual soul cannot be incarnated before the implantation process (Congregation calls it “nidation”). But as I have noted, today’s supporters of delayed animation (e.g., Ford [16]) claim that an embryo is animated just after the implantation (but not much later). Today, no Catholic maintains, as Thomas Aquinas did and as many others did until the 19th century, that a fetus is animated by an intellectual soul 40 days after conception (in the case of a male fetus) and 90 days (in the case of a female fetus). Since abortions are performed after implantation, the ANU type argument discussed by Jackson and Smith cannot be applied today by any “group of Catholics” to abortion (if there are Catholics who do not oppose early abortion, they probably have other justifications). This is the main reason why I concentrate not on the problem of abortion, but on the permissibility of acts that lead to the destruction of early embryos, and above all on research on human embryonic stem cells (since they are harvested from blastocysts 4–5 days after fertilization) and other moral issues concerning early embryos (e.g., the fate of spare embryos after in vitro fertilization, “morning-after” pills, or even some contraceptives).

In this context, it is surprising that no one (as far as I know) has analyzed the ANU type argument in detail in the context of the permissibility of embryo research. A few bioethicists or philosophers have noticed the possibility that the pro-life view on embryo research can be interpreted this way, but their analyses are often oversimplified.

For example, David Martin Shaw, who discusses a version of the ANU in two short paragraphs (he calls it “the argument from doubt”) of his paper about embryo research correctly, notices that the argument is often presented as an analogy: “if I am hunting with a rifle, and I see something move in the trees but am unsure whether it is a deer or a person, I am obliged not to shoot until I establish that it is in fact a deer: better safe than sorry” [32, p. 219]. He is right that this is the traditional way of describing the problem of factual doubts by Catholic moral theologians, but he is mistaken in other respects. He says, firstly (and mistakenly, in my opinion), that the main problem with this argument is that it conflates empirical ignorance with conceptual uncertainty (he borrows the terms from Alex Mauron): we can resolve empirically whether or not the target is a person, but we cannot settle in the same way the issue of the ontological and moral status of embryos. This is certainly true, but it seems that the possibility and the method of resolving the uncertainty does not explain fully the difference between empirical ignorance and conceptual uncertainty: the correctness of a decision procedure under uncertainty (or ignorance) does not depend on whether it is possible to check the accuracy of this decision afterwards. For example, the correctness of the hunter’s decision to shoot or not depends on his evidence at the moment of shooting, not on the future possibility of checking whether the moving entity in the trees was a deer or a person (this problem is related to the discussions about objective and subjective—or “decision”—oughts; see, e.g., [30, pp. 268–271]). Secondly, Shaw says that the hunter should not shoot until he is sure that the target is not a person. Strictly speaking, the hunter will never have this kind of certainty at the moment of shooting; for example, he cannot rule out that some rival has not added hallucinogenic drugs to his morning coffee. Normally, we are allowed to proceed in these types of cases only if the risk of killing a person is negligible.

Kevin Elliott, in a footnote to his article, distinguishes two different ways in which one might defend a pro-life view with an argument based on the “paradox of the heap” [33]. The first is grounded in metaphysical or theological arguments. For example, someone may believe in substance ontology and claim that after the fertilization, all changes that an embryo undergoes are merely “accidental” as opposed to “substantial.”

The second is based on something like the ANU: a cautious response to uncertainty about the moral status of the embryo. However, in describing this argument, Elliott conflates uncertainty or moral fallibility with disagreement. He says that “there is often significant disagreement about the strength of the premises for these metaphysical or theological arguments,” and he maintains that for some pro-life thinkers, this disagreement is a reason for acting cautiously. It is true that there is disagreement about the moment of ensoulment among theologians. But the problem is that disagreement about moral issues among different scholars can exist even if every party is completely certain about his or her moral views. The ANU in my interpretation aims to be a model not only for decision-making under disagreement, but also for decision-making under uncertainty by fallible agents whose credences are divided between different moral doctrines (another short discussion of an argument similar to the ANU can be found in [34]).

## The critique of the ANU

Now, I will demonstrate that the pro-life view, even in my interpretation, which is seemingly stronger than the standard one, has limited scope. It cannot be used to restrict promising research on human embryos, but it does have some interesting applications. I am going to focus on two problems, one general and one more specific: the first concerns the possibility of adapting absolutist moral doctrines to the decision theory framework; the second concerns intertheoretic value comparisons.

In the third section of this article, I stated that the ANU makes use of the decision theory model of moral reasoning in situations of normative uncertainty. One could claim that my description of the ANU is mistaken, because pro-life views are usually based on a deontological ethical framework that treats right actions as a matter of complying with rules and evaluates outcomes only in terms of the actions that produced them. Therefore, one could argue that this framework does not fit the decision theory framework which can be used only by consequentialist theories. But this does not have to be the case. Ethical doctrines usually give clear recommendations in situations where the results of actions and all morally relevant information are known [35]. For example, they disagree on what to do in so-called trolley cases: if you flip the switch, then you know one person will be killed for sure, whereas if you do not flip it, then five other people will be killed. In these kinds of cases, you are sure that they will be killed and you are certain that they are persons. But what to do when there is no such certainty about the results or—as in the case of embryo research—about the ontological status of some beings? Since deontology, like every other normative doctrine, must deal with uncertainties, it can be accommodated—as several authors have recently observed—“in something like the standard decision-theory framework, and thus expected utility theory need not be thought of as a tool available only to the consequentialist” [36, p. 525].

Nevertheless, the decision theory model, when used to guide our action in moral terms and within the deontological framework, is highly controversial. It assumes that in the face of risk, we are rational if and only if we maximize expected value (whatever it is). The ANU assumes a similar model for an agent who wants to act morally in the face of uncertainty. When an agent wants to make morally correct decisions, she must obey the same principles of rationality that she should obey in any other kind of decisions. Therefore the ANU assumes a meta-moral obligation of maximizing expected moral value: this is not only a requirement of rationality, but also a requirement of morality. If one wants to act morally in situations of normative uncertainty, one should follow the rules of rationality as they are understood by the decision theory model.

The main problem that the supporters of the ANU have to deal with consists in making intertheoretic comparisons of values. Normally, when we use an expected utility calculus, we use a common scale by which we can measure the values attached to these different outcomes (this is one reason why so many examples refer to money). But there does not seem to be anyway of making this kind of intertheoretic comparison of moral values between different theories or doctrines. The moral theories themselves cannot be a starting point since we need some meta-theoretical tool to judge which action has the highest expected moral value in situations where our credence is divided between different moral theories (or between different views on the moral status of a human embryo).

Let me demonstrate this problem with the example of destructive embryo research. The followers of the ANU try to argue that we are dealing, on the one hand, with the doctrine that ascribes a very high value to the embryos and, on the other, with theories that claim that destroying embryos does not matter from the moral point of view (for example, because early embryos are like normal human tissues). So, they argue, we should aim the safer course. Unfortunately for the pro-life view this is not a proper description of the controversy. On the one hand, the supporters of the pro-life view indeed claim that since it is possible that early embryos have full moral status and no one can be sure that it is not so, everyone should treat embryos as morally equal to adult human beings. But on the other hand, the supporters of destructive human embryo research claim that embryo research may help many future people, either living now or those who have not been born yet—that it may lead to the development of therapies that lengthen lives, alleviate suffering, and allow parents to achieve their reproductive goals. Fervent supporters of human embryonic stem cell (hESC) research write: “Failing to pursue this research could result in thousands, perhaps millions, of avoidable deaths, not to mention great pain and suffering” [37, p. 307]. Moreover, embryo research also provides an opportunity to conduct basic research on the process of human development. Therefore, according to the supporters of destructive human embryo research, it would be extremely wrong to abandon this kind of research and the option of abandoning research would have a very negative value.

How can we compare these two positions? How are we to “calculate” which option has higher expected moral value? This is not an easy task because we have to deal with at least two competing views which use different hierarchies of values. On the one hand, if one of the pro-life views is correct, then embryo research is strictly impermissible because embryos have full moral status. This is because they assign either infinite or extremely high disvalue to intentionally killing an entity of full moral status and only finite (or moderately high) disvalue to allowing people to die (let us assume that these deaths could be avoided by the therapies resulting from embryo research). This means that pro-lifers can agree that the expected benefits from embryo research could be enormous, but not so large as to justify killing entities with full moral status. On the other hand, if one of the pro-research views is correct, abandoning embryo research is very wrong, because we are missing a chance to save thousands of future people and we do not sacrifice anything of great moral significance by destroying human embryos in the process.

The proponents of the ANU would have to show, firstly, that their argument works in situations in which we are dealing with at least two competing normative views about the moral status of early embryos, and secondly, that abandoning destructive human embryo research would indeed have higher expected moral value than continuing this kind of research. I claim that the followers of the ANU do not reach this second point: it is impossible to compare expected losses and benefits of doing or abandoning embryo research from a point of view which is external to any moral doctrine.

In recent literature, I have found one serious attempt to describe a tool that could help in making intertheoretic comparisons of values. This is the “Principle of Equity among Moral Theories” (PEMT), which claims that “the maximum degrees of moral rightness of all possible actions in a situation according to competing moral theories should be considered equal” [23, p. 84] (the same is said about “the minimum degrees of moral rightness”). Unfortunately, this idea seems to be obviously mistaken because different moral views can strongly disagree about the moral value of the same action: they can disagree about how wrong or how right an action is. In the example discussed in this article, we are uncertain about which of two views on experiments on early embryos is correct: the first says that destroying embryos for research is strictly forbidden because they have as high a moral status as adults; the second says that research on embryos is not forbidden, but morally demanded, because the moral status of embryos is so low that the expected future gains can easily override the disvalue of embryo destruction. The PEMT cannot help to solve this dilemma because it would say that in this situation the worst possible action according to the first normative view (destroying embryo for research) should be considered equal to the worst possible action according to the second view (abandoning embryo research). The problem is that in the first case, we would do something gravely wrong (according to this normative doctrine): we would be killing large numbers of beings that have moral status equal to the status of adults to gain some uncertain future scientific benefits; therefore, we are committing one of the most morally horrible acts. However, in the second case we are doing something wrong, but probably not so gravely wrong within this normative view (there are many much worse categories of actions than abandoning destructive embryo research in this normative framework). Therefore, the PEMT is not the tool we are looking for: it cannot help in comparing different values according to different moral doctrines since it equalizes these values by definition (for more objections, see [38]).

Nevertheless, there are cases in which the ANU type of reasoning works perfectly well. Suppose, for example, that John must decide whether to kill some living organism or not (this is a modified version of the example discussed by [24]). John believes that it is highly probable that from the moral point of view it does not matter if he kills this type of being or not, but he is not absolutely certain of his normative views (let us assume that his degree of credence is 0.99). This means that he thinks that there is a very small chance that killing this kind of organism is in fact morally wrong (his degree of credence regarding this view is 0.01). Therefore, given the view that is the most probable in his opinion, it would be neutral from the moral point of view to kill or not to kill this organism. But given the alternative view, it would be morally wrong to kill this type of organism and morally good not to kill it. If John wants to maximize the expected moral value of his decisions, he should choose not to kill this organism, even though he believes that the probability that killing this type of being is morally wrong is indeed extremely low. The application of the ANU in this type of reasoning seems to be correct, because here there is no problem with intertheoretic comparisons of values. John does not have to compare any values or disvalues between different moral doctrines or views on the moral status of this living organism, because one view says that everything he does in the situation is morally neutral. In these types of cases, the ANU would indeed give one a reason to prefer the “morally safer” option, only if the probability that this option is morally correct is greater than zero (but see two recent critiques of this kind of argumentation [39, 40]).

The above example has an interesting implication in the case of early embryo protection: the ANU would in fact prohibit destroying early embryos in situations in which there are no prospects for achieving anything of moral worth (e.g., destroy them just “for fun”). Nevertheless, the decision theory model of moral reasoning in situations of normative uncertainty is a dangerous ally of the pro-life position. At first, the ANU seems to help to defend pro-life views in the case of embryo research because it promised to reconcile the uncertainty about the ontological status of embryos with the certainty about normative obligations, and was based on a cautious response to the ambiguity about the ontological status of early human embryos. However, the ANU does not withstand closer inspection, and it turns out that the supposed ally of the pro-life position cannot be used to justify limiting promising research on early embryos.

## Footnotes

1 In this article, the term “embryo” refers to the entity that precedes a human being until the time the primitive streak starts to develop (about 2 weeks after fertilization).

2 Jackson and Smith distinguish “decision-ought” from “objective-ought”: the first takes into consideration the epistemic state of an agent.
